# A rare case of concomitant endometrioid adenocarcinoma arising from uterine adenomyosis and clear cell carcinoma arising from parametrial deep endometriosis

**DOI:** 10.1186/s12905-024-03170-4

**Published:** 2024-08-02

**Authors:** Cailu Zhou, Xiaojing Luo, Mengjie Tang, Fangyuan Luo, Zhi Liao

**Affiliations:** 1 Department of Obstetrics and Gynecology, Affiliated Hospital of Southwest Medical University, Luzhou, 646099 China; 2 University of Electronic Science and Technology of China, Chengdu, 610054 China; 3 Department of Obstetrics and Gynecology, Sichuan Provincial People’s Hospital, Chengdu, 610072 China

**Keywords:** Adenocarcinoma, Adenomyoma, Endometriosis, Case report

## Abstract

**Background:**

Carcinomatous changes from the ectopic endometrial glands in endometriosis have been reported in many studies, but malignant transformation from uterine adenomyosis/adenomyoma is rare. And clear cell-like adenocarcinoma represents a seldom-encountered malignant pathological variant of ectopic endometrium.

**Case presentation:**

This case report presents a case of a 44-year-old nulliparous woman begun with abdominal pain and intestinal obstruction. Past medical history showed laparoscopic ovarian endometriotic cyst excision. Ultrasound indicated adenomyoma and a parametrial hypoechoic nodule with abundant blood flow signals and unclear boundaries. Deep invasive endometriosis was considered preoperatively. The patient underwent laparoscopic subtotal hysterectomy and bilateral adnexa resection. Chocolate cyst-like lesion was observed in the parametral lesion. Postoperative pathological examinations suggested endometrioid adenocarcinoma arising from eutopic endometrium and adenomyoma. Ectopic endometrium in the myometrium combined with atypical hyperplasia and formation of endometrioid adenocarcinoma. Left parametrial lesions suggested poorly differentiated endometrioid adenocarcinoma combined with clear cell carcinoma. CD10 + endometrial stromal cells were observed surrounding tumor cell masses. Combined with surgical founding and pathological characters of the left parametrial adenocarcinoma, the parametrial lesions were more likely to be carcinomatous changes of the original deep endometriosis.The patient underwent subsequent transabdominal tumor cell reduction surgery and chemotherapy.

**Conclusion:**

We herein present a rare case of combined endometrioid adenocarcinoma arising from uterine adenomyosis and clear cell carcinoma arising from parametrial deep endometriosis that may help inspire additional studies in the future. The patient underwent robot-assisted laparoscopic subtotal hysterectomy, bilateral adnexa resection, deep endometriosis lesion resection and bilateral ureteral stent placement. Following surgery, a chemotherapy regimen of Taxol and Carboplatin was administered.

## Background

Endometriosis is a chronic benign disease and its malignant transformation rate is approximately 1% [1]. The most common site of endometriosis malignancy is ovary. Other locations also include the intestine, abdominal wall, vagina, cervix, bladder, ureter, pelvic floor muscles, and recto-vaginal septum [2–7]. Malignant transformation from adenomyosis is extremely rare [8–11]. Approximately 0.5–1% of endometriosis cases are complicated by tumors. The pathogenesis likely involves oxidative stress-induced DNA damage, persistent antioxidant production, and somatic mutations in genes such as ARID1A, PTEN, and PIK3CA. Endometrial cancer with coexistent adenomyosis often has a better prognosis, though this cannot currently be explained by molecular characteristics. Studies indicate that 0.14–2.9% of endometriosis patients develop endometriosis-associated ovarian cancer, particularly clear cell ovarian carcinoma, which accounts for 5–12% of cases and typically leads to a poor prognosis. Atypical endometriosis, as a precancerous lesion, aids in the detection and prevention of this disease [12–15]. The most common malignant pathological type in ectopic endometrium is endometrioid adenocarcinoma. Other pathological types also include clear cell-like adenocarcinoma, squamous cell carcinoma, endometrial stromal sarcoma, and Mullerian carcinosarcoma [16–18]. This case presented one rare case of carcinomatous changes of both uterine adenomyoma and deep endometriotic lesions that induced hydronephrosis and intestinal obstruction. We had received the signed informed consent from the patient for this case report and publication and patient anonymity was preserved.

## Methods

A case of cerebral infarction with adenomyosis is reported, and a comprehensive systematic literature search using the PubMed database was conducted. We had received the signed informed consent from the patient for this case report and publication and patient anonymity was preserved.

## Case presentation

**Fig. 1:**
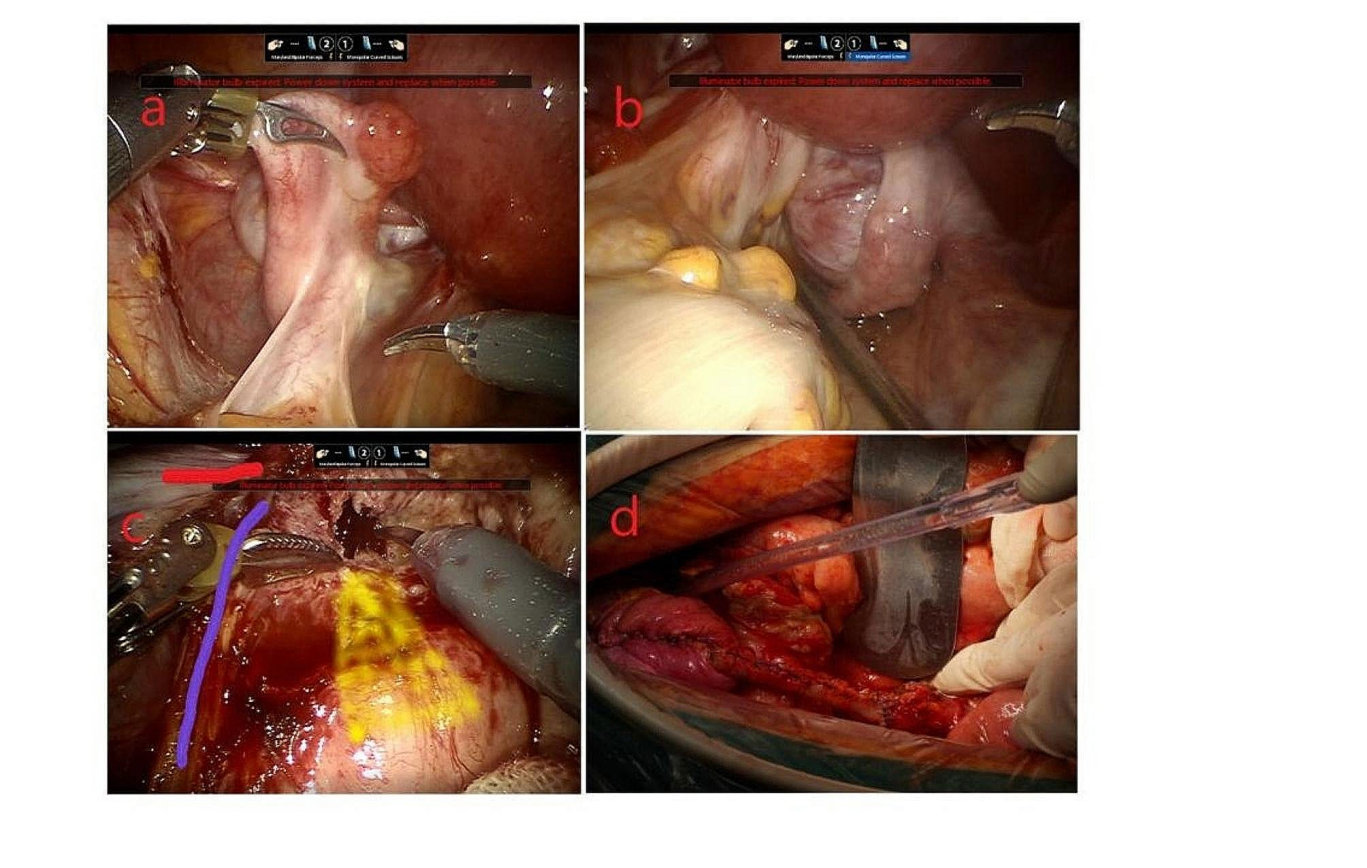
a/b. The outer appearance of endometriosis lesions and the left parametrial lesions in left adnexa; c. chocolate-like fluid in the lesion( red line indicated he proper ligament of the left ovary, purple line indicated left ureter, and the yellow area indicated rectum); d. Bladder valvuloplasty after partial ureterectomy

**Fig. 2:**
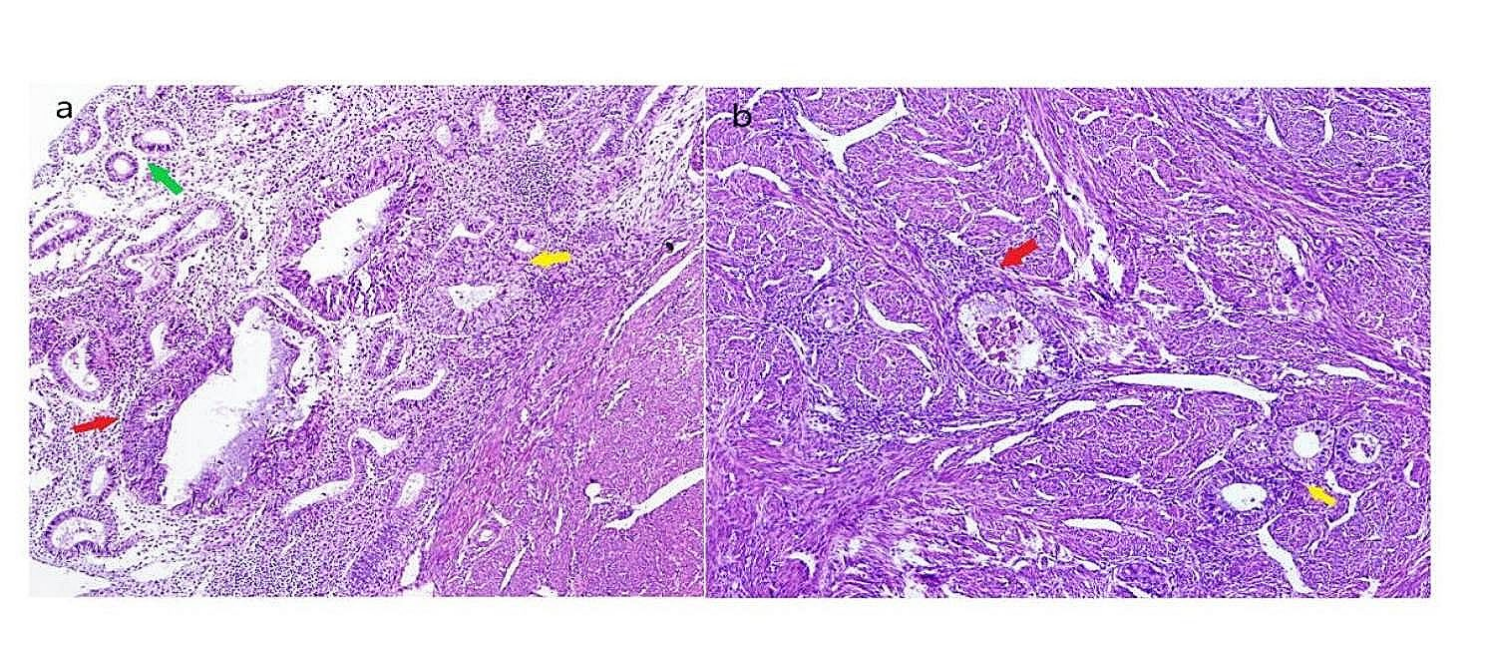
Malignant transformation of endometrial glands in eutopic endometrium and ectopic endometrium of adenomyoma (green arrow indicated normal gland, yellow indicated atypical hyperplasia, and red showed carcinomatous gland)

**Fig. 3:**
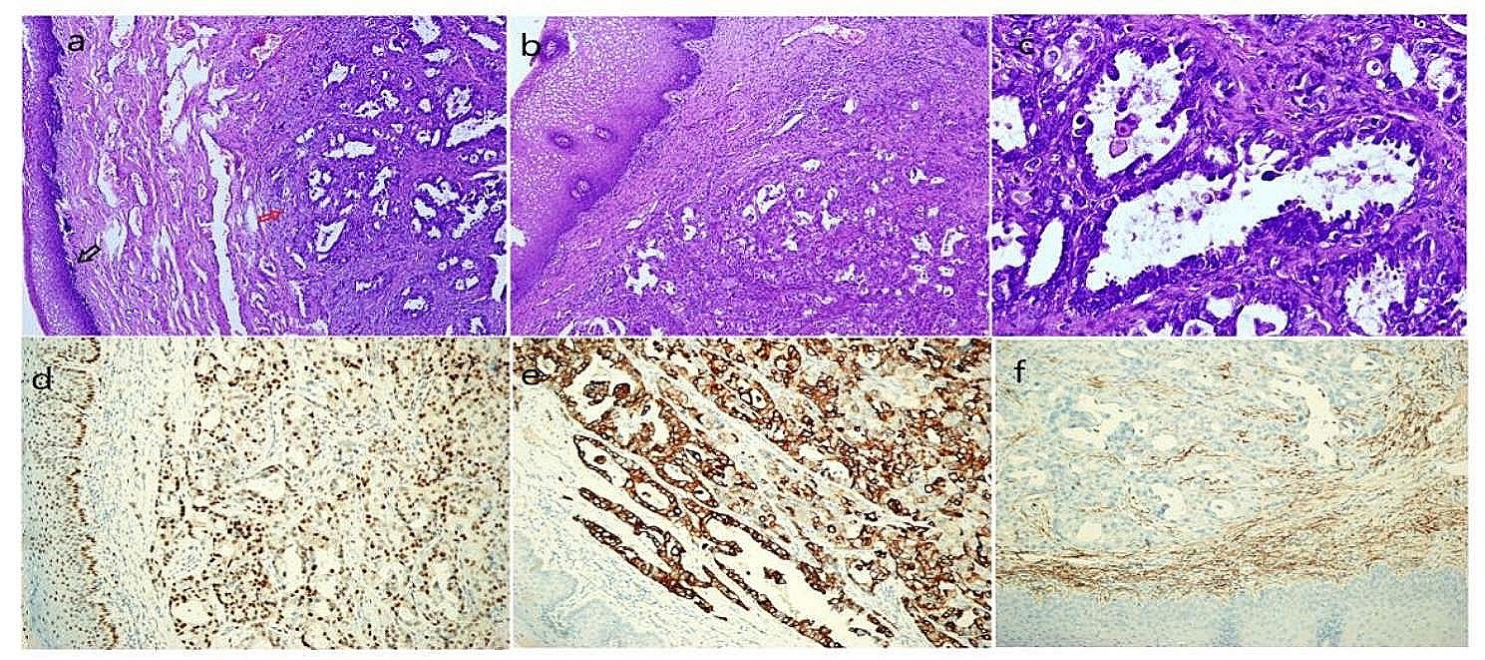
Left parametrial endometrioid adenocarcinoma combined with clear cell differentiation and the immunohistochemical staining. a/b. Endometrioid adenocarcinoma with clear cell components (red arrow) at the vagina wall near the parametrial location (black arrow); c. clear cell carcinoma: hobnail-like cancer cells in glands were observed; d. ER+; e. CK7+, f. adenocarcinoma cells surrounded by CD10 + endometrial stromal cells

The patient was a 44-year-old Chinese woman with G1P0 + 1 and body mass index of 19. She sought treatment in our emergency surgery department due to sudden onset of intermittent abdominal colic with vomiting for 1 day after eating indigestible food. The patient also had the symptoms of intestinal obstruction such as nausea, anal pendant expansion, and no gas nor defecation but did not have discomforts such as fever, bloody stool and vaginal bleeding. The patient underwent laparoscopic cyst excision due to ovarian endometriotic cysts 7 years ago and laparoscopic separating surgery due to infertility 4 years ago. The patient had regular menstrual periods and secondary infertility for 20 years. She received one in vitro fertilization and the embryo transfer 4 years ago; however, embryo implantation failed. Physical examination discovered a 2 cm ovarian cyst 1 year ago but the patient did not receive treatment and follow up. The patient did not have a history of long-term exposure to estrogen, oral short-acting contraceptives, or other drugs. She had no known family history of cancer. Abdominal examination suggested lower abdomen tenderness without rebound pain and no palpable abdominal mass. Gynecological examination revealed smooth cervical appearance, uniformly enlarged uterus with a size close to the size of a 2-month pregnancy, left adnexa had patchy thickness, and left parametrial tissues had a palpable hard and irregular nodule of approximately 3 cm that involved left vaginal fornix. Gynecological color ultrasound indicated that the endometrium was 0.6 cm. The thickness of the anterior and posterior walls of myometrium was not even, myometrial echo was coarse and uneven especially the posterior wall, and there was an echo from a mass with unclear boundaries at the size of 3.9*3.3 cm. The posterior cervical wall had a detected hypoechoic nodule at the size of 1.7*1.5 cm, there were abundant blood flow signals, and the boundaries were clear. The renogram showed the curve of left renal incomplete obstruction. The transrectal contrast-enhanced ultrasound examination detected a solid hypoechoic mass of approximately 1.8*3.5 cm in posterior cervical wall; the mass had irregular morphology and unclear boundary, the mass invaded the anterior wall of rectum through the rectouterine pouch. The color Doppler flow imaging suggested that there were more abundant blood flow signals in the hypoechoic mass. Gastroscopy and colonoscopy did not show obvious abnormality. The patient had normal liver and kidney functions, CA-125 was 183.4U/ml, and CA-199 was 66.7U/ml. Therefore, adenomyosis combined with adenomyoma and deep endometriosis combined with ureteral obstruction and intestinal obstruction were considered. The patient underwent robot-assisted laparoscopic subtotal hysterectomy, bilateral adnexa resection, deep endometriosis lesion resection and bilateral ureteral stent placement in the Department of Genecology of our hospital. The surgery showed that uterus enlarged to the size of approximately 2 month pregnancy, the appearance of bilateral oviducts did not have obvious abnormality, the surface of left ovary had brown endometriotic lesions, the right ovary was swelling with a diameter of 3 cm and had adhesion with the posterior wall of the uterus and rectum, and the proper ligament of the left ovary had dense adhesion with left ureter, left posterior wall of uterus, rectum (Fig. 1a/b). There was a 3 cm hard lesion with unclear boundaries in the adhesion area that involved left vaginal fornix, lower segment of left ureter, and rectum. There was a chocolate cyst-like lesion of 1 cm that contained chocolate-like old bleeding fluid (Fig. 1c). The lower segment of left ureter at length of 10 cm had thickened and was hard and the segment of rectum at approximately 4 cm behind cervix had thickened and hard muscular wall. There was no lesion involvement in the upper abdomen. Dissection of endometrium after hysterectomy did not show obvious lesions, the myometrium was diffuse and thickened, and the posterior wall of uterus had an adenomyoma-like lesion of approximately 4 cm. Postoperative pathological examinations suggested eutopic endometrium had multifocal atypical hyperplasia of endometrial glands and formations of highly/moderately differentiated endometrioid adenocarcinoma. The maximum diameter of intramyometrial adenomyosis was 5 cm and most gland components exhibited atypical hyperplasia and highly/moderately differentiated endometrioid adenocarcinoma. Other myometrium wall tissues were scattered in the ectopic endometrium tissues combined with atypical hyperplasia and formation of endometrioid adenocarcinoma (Fig. 2). Left parametrial lesions suggested poorly differentiated endometrioid adenocarcinoma combined with partial clear cell carcinoma involvement (Fig. 3). Uterine specimens showed tumor thrombus in vessels and perineurium invasion. There was no tumor involvement in cervix. Left ovary had endometriotic cysts with no tumor involvement, and right ovary had tumor involvement. Immunohistochemistry results of left parametrial lesions suggested ER(+)PR(+); CK7(+); CD10(-); CEA(+); Ki67 (50%)(Fig. 3). Immunohistochemistry results of adenomyosis suggested ER(+); PR(+); CK7(+); CD10(-); CEA(focal+); Ki67(20%). The patient was considered to have carcinomatous changes of both uterine adenomyoma and deep endometriotic lesions. On 8th day after the first surgery, the patient underwent transabdominal tumor cell reduction surgery including greater omentum resection, appendectomy, pelvic lymph node dissection and para-abdominal aortic lymph node dissection, partial rectectomy, intestinal anastomosis and resection of the lower segment of left ureter, left ureteral cystoplasty and left ureteral stent implantation. Postoperative pathological examination suggested that ureter had tumor infiltration, bilateral pelvic lymph nodes and para-abdominal aorta lymph nodes all had tumor metastases, tumor infiltration was observed on the serosal surface of the intestinal wall of of partial rectum, the muscular layer of intestinal wall had tumor infiltration, and the appendix and greater omentum did not have tumor infiltration. After a satisfactory postoperative recovery, the patient was treated with Taxol and Carboplatin chemotherapy regimen. After chemotherapy completion, the patient has been undergoing regular follow-up examinations; no recurrence has been noted at 18 months.

## Discussion

In 1959, Colman and Rosenthal modified Sampson’s criteria to apply to carcinomas developing from adenomyosis: (i) carcinoma should be absent from the normally situated endometrium and anywhere else in the pelvis; (ii) the carcinoma should be actually observed to be arising from the epithelium of the areas of adenomyosis and not invading from another source; and (iii) endometrial stromal cells should surround the aberrant glands to support a diagnosis of adenomyosis [17]. In this case, Co-existence of normal endometrial glands, atypical hyperplastic glands, and cancerous glands were observed both in eutopic endometrium and ectopic endometrium in uterine adenomyoma lesions. In addition, obvious endometrial stromal cells surrounding cancerous glands were observed. These observations were sufficient to prove that eutopic endometrium and ectopic endometrium in myometrium both had malignant transformation and they were both primary. Carcinomas arising within adenomyosis exhibit an exceptionally low incidence rate, with only 1% of cases developing malignant transformation. Although the precise mechanism remains unclear, oxidative stress, inflammation-induced alterations in the microenvironment, and mutations in multiple genes may all contribute to this process [19].

In reported cases of carcinomatous change of uterine adenomyoma or adenomyosis, the probability of simultaneous malignant transformation of eutopic endometrium was approximately 20% [9, 11]. Bingjian Lu and colleagues reported 3 cases of serous carcinoma in uterine cervical adenomyosis. Among them, one case did not have carcinomatous changes, one case had serous carcinoma, and one case had endometrioid adenocarcinoma in eutopic endometrium [9]. Adenomyosis often coexists with other pathological changes, particularly estrogen-related disorders such as endometrial polyps, uterine fibroids, and endometrial cancer, which are frequently associated with adenomyosis. The likelihood of endometrioid adenocarcinoma associated with adenomyosis ranges from 18 to 66%. It is noteworthy that carcinomas arising within adenomyosis and carcinomas associated with adenomyosis are two distinct entities. Atypical hyperplasia, often regarded as a precursor to cancer, is typically absent in ectopic endometrial tissue associated with the latter condition, indicating insufficient evidence for continuous malignant transformation [20]. However, the presence of atypical hyperplasia in the myometrium in this case suggests a closer correlation with the carcinomas originating from adenomyosis.

The most important feature of this case was that whether the poorly differentiated endometrioid adenocarcinoma in the left parametrial area combined with partial clear cell carcinoma was the carcinomatous changes of the original deep endometriosis or carcinomatous changes and metastasis of uterine adenomyoma or eutopic endometrium. HE staining showed that the structure and morphology of parametrial tumor cells was different from those of adenomyosis and tumor cells in eutopic endometrium. Because normal endometriotic glands surrounding parametrial lesions or in lesions were not found, diagnostic criteria of carcinomatous changes of endometriosis proposed by Sampson in 1925 could not be completely met [21]. Therefore, the possibility that this lesion was from metastasis could not be ruled out. Normal endometrial glands could not be found in left parametrial adenocarcinoma that had involvement of rectum, ureter, and right ovary. Thus, the possibility of metastasis of carcinomatous change of adenomyosis to left parametrial was more likely.

However, combined with surgery and pathological evidence, we considered that the left parametrial adenocarcinoma was more likely to be carcinomatous changes of the original deep endometriosis. First, the left parametrial lesion was generally not adjacent to the adenomyoma lesion and the left parametrial adenocarcinoma lesion contained chocolate-like fluid, indicating that this site was likely to have the original deep endometriosis. Next, tumor cells in parametrial adenocarcinoma lesions and endometrial tumor cells in endometrium and adenomyosis had significant differences at differentiation level, structure and morphology. In addition, there were CD10 + endometrial stromal cells surrounding tumor cell masses in parametrial lesions, indicating that tumor cells were from endometrial glands. Finally, normal endometriotic glands were not found in left parametrial adenocarcinoma lesions; which might be associated with insufficient or biased material collection in pathological examination or carcinomatous change of all endometriotic glands in that place.

Immunohistochemical staining of microsatellite instability in left parametrial adenocarcinoma lesions that had clear cell components and eutopic endometrioid adenocarcinoma was performed. MLH1, MSH2, MSH6, and PMS2 were all positive in parametrial lesions, whereas PMS2 was negative in eutopic endometrioid adenocarcinoma lesions. The microsatellite instability levels between these two were not consistent, indicating that the pathogenic mechanisms of these two carcinomatous changes might be different and the parametrial lesion was likely to be the carcinomatous change of the original deep endometriosis. Currently, cases of cancerization of deep endometriosis are rarely reported. Its main clinical manifestations include progressive dysmenorrhea, chronic pelvic pain, dyspareunia, and urinary and gastrointestinal symptoms, with or without abnormal uterine bleeding. Baoxuan Li et al. reported a case of deep infiltrating endometriosis malignantly invades the cervix wall and rectum wall, presenting primarily with hypogastralgia, diarrhea, and intermittent fever [22]. Conversely, cancerization of adenomyosis typically presents with abnormal uterine bleeding and pelvic pain [23]. Notably, pain is the most common symptom of extrauterine adenomyoma, occurring in 56% of cases. A history of endometriosis, adenomyosis, or uterine tissue morcellation may suggest this condition. Pathological diagnosis can aid in differentiation [24]. It is considered that obesity and unopposed estrogen use are high-risk factors leading to carcinomatous change of endometriosis.However, the patient did not have the above high risk factors and did not have a family history. The eutopic and ectopic endometrium both had carcinomatous changes, suggesting that the genetic factor of the patient might play a critical role in disease pathogenesis.

For the early treatment of endometriosis, some scholars argue that early surgical intervention can eradicate the initial disease and prevent complications, for example, the malignant transformation of endometriosis.Additionally, endometriosis may act as a protective factor against endometrial cancer; the risk of death is halved in endometrial cancer patients with coexistent adenomyosis compared to those without adenomyosis [22, 25].There is still controversy about the adjuvant treatment of carcinomatous change of endometriosis after surgery. Some scholars consider that radiotherapy can benefit patients more than chemotherapy. However, considering the ovarian and colorectal involvement in this patient, chemotherapy was chosen. This patient had a history of endometriotic cysts and dysmenorrhea but did not have menstrual disorders. Therefore, it was diagnosed as deeply infiltrating endometriosis before surgery. Tumors were discovered in pathological examination after hysterectomy. However, retrospective analysis of preoperative examination showed that the patient had CA125 of 183.4U/ml and color ultrasound suggested abundant blood flow in the posterior cervical wall lesion. These results all suggested the possibility of carcinomatous changes.

## Conclusions

The treatment experiences on this patient were summarized. For patients suspected to have adenomyosis combined with deep invasive endometriosis, physicians should be alert for physical signs and features of ectopic endometrial malignant transformation such as obesity, diabetes mellitus, history of unopposed estrogen use, abnormal uterine bleeding symptom, CA125 over 200 U/ml, and abundant blood flow signals in lesions suggested by color ultrasound should be cautious. Early surgical diagnosis and satisfactory tumor reduction surgery are the keys to treatment. However, the mechanisms underlying the malignant transformation of endometriosis remain unclear and warrant further investigation.

## Data Availability

The data supporting the conclusions of this article is available from corresponding author.
