# Correction: A rare case of concomitant endometrioid adenocarcinoma arising from uterine adenomyosis and clear cell carcinoma arising from parametrial deep endometriosis

**DOI:** 10.1186/s12905-025-03603-8

**Published:** 2025-02-20

**Authors:** Cailu Zhou, Xiaojing Luo, Mengjie Tang, Fangyuan Luo, Zhi Liao

**Affiliations:** 1 Department of Obstetrics and Gynecology, Affiliated Hospital of Southwest Medical University, Luzhou, 646099 China; 2 University of Electronic Science and Technology of China, Chengdu, 610054 China; 3 Department of Obstetrics and Gynecology, Sichuan Provincial People’s Hospital, Chengdu, 610072 China

**Correction: BMC Women's Health 24, 440 (2024)**

**https://doi.org/10.1186/s12905-024-03170-4**

In this article [1], the affiliation details for the authors Cailu Zhou and Zhi Liao were incorrectly given as ‘Southwest Medical University, Luzhou 646099, China’ but should have been ‘Department of Obstetrics and Gynecology, Affiliated Hospital of Southwest Medical University, Luzhou 646099, China’.

The original article has been corrected.
